# A preclinical numerical assessment of a polyetheretherketone femoral component in total knee arthroplasty during gait

**DOI:** 10.1186/s40634-017-0078-4

**Published:** 2017-02-01

**Authors:** Lennert de Ruiter, Dennis Janssen, Adam Briscoe, Nico Verdonschot

**Affiliations:** 1 Orthopaedic Research Laboratory, Radboud University Medical Center, Radboud Institute for Health Sciences, P.O. Box 9101, 6500 HB Nijmegen, The Netherlands; 2 Invibio Ltd, Thornton Cleveleys, Lancashire FY5 4QD UK; 3 Laboratory for Biomechanical Engineering, University of Twente, Enschede, The Netherlands

**Keywords:** Total knee arthroplasty, Polyetheretherketone, Finite element analysis, Implant mechanical integrity, Stress shielding

## Abstract

**Background:**

Conventional total knee replacement designs show high success rates but in the long term, the stiff metal components may affect bone quality of the distal femur. In this study we introduce an all-polymer total knee replacement device containing a PEEK femoral component on an UHMWPE tibial implant and study its mechanical integrity, fixation, and stress shielding of the periprosthetic femur.

**Methods:**

The implant was analysed in finite element simulations of level gait, adopted from the ISO 14243-1 standard. Mechanical integrity of the implant and underlying cement mantle were tested, and the fixation strength of the cement-implant interface was studied. Stress shielding was assessed based on strain energy density distributions in the distal femur. We compared PEEK and CoCr implants for mechanical performance and fixation, and compared both versions against an intact case to determine the change in bone strain energy density.

**Results:**

The mechanical integrity of the PEEK and CoCr components was similar in magnitude, but differences in stress patterns were found. Moreover, the cement mantle was loaded more heavily in the CoCr configuration. Under similar interface properties, the CoCr-cement interface was more at risk of failure than the PEEK-cement interface. The bone strain energy density distribution of the PEEK implant was similar to the intact case, while the CoCr implant showed signs of stress shielding, and a different distribution than the intact and PEEK models.

**Conclusions:**

During gait, the PEEK femoral component performed similarly to CoCr, with no added risk for the cement mantle. The reduction in stress shielding for PEEK was evident and confirms the potential reduction in long-term loss of bone stock for this all-polymer knee implant.

## Background

Total knee arthroplasty (TKA) is a highly effective procedure for patients who suffer from severe chronic knee pain and injury. In the USA and Europe combined, over one million implantations are performed every year, which have high survival rates of up to 97% after 10 years (AOA 2013; Niinimäki et al. 2014; NJR 2013; SKAR 2013; Victor et al. 2014). Despite its success, failure of the reconstruction is still a matter of concern. Most causes for revision can be summarized in five failure modes: aseptic loosening, infection, polyethylene wear/osteolysis, instability, and periprosthetic bone fractures (Kasahara et al. 2013; Le et al. 2014; Schroer et al. 2013). Literature is not always in agreement about the underlying mechanisms for these types of failure. At least three of these failure modes can be induced (not exclusively) by stress shielding. As bone turnover is regulated by mechanical loading, stress shielding will eventually lead to a loss of bone stock (osteolysis/resorption), which in turn can lead to aseptic loosening of the implant or, to a lesser extent, can weaken the bone such that it will fracture (Lavernia et al. 2014; Van Lenthe et al. 2002). Additionally, revision surgery is complicated by poor bone stock, even if this is not the reason for revision.

One possible solution to these problems is the use of more compliant materials that allow loads to be distributed more physiologically over the periprosthetic bone. Although the effects of a more compliant implant material have hardly been studied, a small number of studies can be found in which the conventional metal-on-polymer was replaced with a polymer-on-polymer design (Bradley et al. 1993; Moore et al. 1998). Both studies describe the use of a polyacetal (Delrin®) femoral implant against polyethylene. During those studies the implants did not show signs of failure through fracture or wear after a minimum follow-up period of 5 years. Nonetheless, the implant was discontinued due to a decreased survival of the proposed implant, as a consequence of infection caused by poor packaging (Bradley et al. 1993; Moore et al. 1998). Although hypothesized in the polyacetal implant study, none have investigated the effect of a low-stiffness implant on the bone stock.

In this study we introduce a novel polyetheretherketone (PEEK) femoral component combined with an ultra-high molecular weight polyethylene (UHMWPE) tibial component. PEEK is generally known for its rigidity, durability and biocompatibility, and has previously been used in spinal and cranio-maxillofacial surgery, upper extremity arthroplasty and fracture fixation devices (Kurtz and Devine 2007). Since the late 1990’s, applications are also considered for total hip replacement (Akay and Aslan 1996; Ghosh and Gupta 2014; Nakahara et al. 2013; Pace et al. 2002; Rankin et al. 2016).

Changing the implant material will also have an effect on the stress distribution in the reconstruction, and could result in local stress intensities in the implant itself and in the underlying cement mantle, potentially causing implant fracture or cement failure. Furthermore, PEEK will bond differently with bone cement, even when the surface features are similar. To that end, fixation at the cement-implant interface needs to be investigated.

Ultimately, new implants and new designs need to be assessed in controlled clinical studies. However, to minimize patient risks extensive pre-clinical evaluations need to be performed.

The aim of this study is to explore the feasibility of using a PEEK femoral component in TKA by assessing a number of aspects related to the implant mechanical integrity and its fixation. Furthermore, we assessed the potential benefit of reduced stress shielding of PEEK relative to metal.

## Methods

**Fig. 1:**
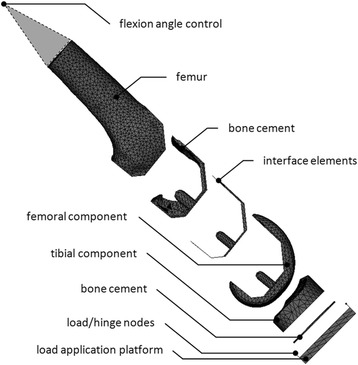
The finite element model and its components

We evaluated a Cobalt-Chromium (CoCr) and PEEK implant using quasi-static finite element simulations (Marc/Mentat 2012, MSC Software Co, Newport Beach, CA, USA) for level walking and adopted the required kinematic and kinetic input for gait from the ISO 14243-1 standard (ISO 2009). The geometry requirements included a load applicator to which the tibial component was attached (Fig. 1). All solid elements were meshed with four-noded tetrahedral elements and an average edge length of 2.5 mm, consistent with convergence testing of a similar model (Zelle et al. 2009). Interface elements were meshed using six-noded cohesive elements.

### Boundary conditions

The applicator was loaded via two hinge joints located on the anterior and posterior side, biased slightly medially to allow for a medial-lateral load distribution. Between these points the varus/valgus axis was situated, around which the applicator was free to rotate. This axis could also move and rotate within the transverse plane, but was restricted in flexion/extension. Together with bone cement and the tibial implant, this part is further on referred to as the tibial construct. Knee flexion was controlled by the femoral construct, consisting of the femoral implant, a layer of bone cement and a distal femur. The knee flexion axis was determined anatomically and fixed during the gait cycle. Medial-lateral translation of the femoral construct along this axis was restricted. These constraints allowed the knee flexion angle to be determined arithmetically and controlled by a node in the hip joint centre, which could move in the sagittal plane. The hip joint centre control node was attached to the proximal end of the distal femur with stiff springs. Degrees of freedom that were restricted in one construct were allowed in the other, enabling any relative tibiofemoral motion to occur.

### Loading conditions

Following the ISO 14243-1 standard, the tibial construct was loaded with three components of load. The first, and most pronounced, was the axial force. This force ran parallel to the tibial axis and represented the ground reaction force. Secondly, an anterior-posterior (AP) force was applied, representing the femoral roll-back/forward mechanism. Finally, a rotational torque was included around the longitudinal axis of the tibia, representing internal/external rotation of the lower leg during gait. Excessive motion resulting from the tibial load application and degrees of freedom was damped by two components. One component limited AP translation, the other limited internal/external rotation, representing ligaments and soft tissues, respectively. Both constraints were formulated as bilinear springs with a stiffness and slack length provided by the ISO 14243-1 standard. The tibiofemoral friction coefficient was set at 0.2 for both CoCr-UHMWPE and PEEK-UHMWPE bearing couples. No frictional data specific for the PEEK-UHMWPE bearing couple is available, hence the parameter was kept the same for both and set slightly high to not underestimate its contribution to the predicted stresses.

The gait cycle, as prescribed by the ISO standard was discretized into 100 increments. Due to low contact forces and a relatively high tibial rotational moment, the congruency of the femoral and tibial components could not prevent numerical instabilities during the swing phase. This was resolved by advancing the peak torque in the swing phase of the gait cycle by three percent, restoring the balance between contact forces and torque. Low contact forces in the swing phase meant this adjustment had no significant effect on the stresses in the model.

### Geometry and material properties

The implant that was used in this study was a right-sided posterior cruciate ligament retaining cemented femoral implant with a cemented all-poly UHMWPE tibial component. The edges of cement pockets in the femoral component were not modelled, nor were the mechanical interlock features on the bottom of the tibial component, to avoid numerical artefacts. The cement layer was modelled with full coverage and a thickness of 1 mm, corresponding to manufacturer specifications.

Material properties for the UHMWPE, CoCr, PEEK and bone cement (polymethylmethylacrylate, PMMA) were provided by the manufacturers of the respective components. Both the geometry and the material properties of the distal femur were obtained from a CT scan of an 81-year old male. The image grey values were linearly scaled between physiological Young’s Moduli for distal femora (0–20 GPa) and assigned to the bone elements (Ashman and Rho 1988; Cuppone et al. 2004; Turner et al. 1999). For evaluating stress shielding, a reference model was used in which the entire femoral construct received material properties from CT data, hence, having the same geometry, yet different material properties (Table 1). To evaluate fixation of the femoral component, the model was expanded with a zero-thickness interface layer between the implant and bone cement. These cement-implant interface elements were given properties that reflected the cohesive behaviour of bone cement and CoCr/PEEK (Table 1). Failure, or debonding/loosening, was determined by providing failure criteria for the interface elements. Both interface properties and failure criteria were obtained experimentally or were taken from literature (Lewis 1997; Murray et al. 1993; Zelle et al. 2011). As PEEK-PMMA interface properties are not available, the same values were used as for the CoCr-PMMA interface, with the exception of shear strength, which was experimentally determined at approximately half the strength of CoCr-PMMA.

**Table 1:** Material properties

Material	Young’s modulus (MPa)	Poisson’s ratio	Yield strength (MPa)^a^
CoCr	210000	0.3	600/600
PEEK-Optima®	3700	0.362	117/90
UHMWPE	1100	0.42	n/a
PMMA	2866	0.3	97/40
Femur	1–20000	0.3	n/a
CoCr-PMMA Interface	5732/57.32/151.36^a^	n/a	70/2.1/8
PEEK-PMMA Interface	5732/57.32/151.36^a^	n/a	70/2.1/4

^a^Compressive/Tensile/(Shear)

### Outcome measures

The model was used to investigate the relative safety of PEEK as compared to CoCr on the femoral implant, bone cement and fixation, and to study the changes in bone remodelling stimuli of the periprosthetic region. The principal stresses were analysed for 1) the femoral component and 2) the cement mantle. Both implant and cement mantle of the CoCr and PEEK constructs were compared to one another to determine relative mechanical safety. Fixation of the implant was analysed at the 3) cement-implant interface using an adopted Hoffman failure index (FI), which is an arithmetic combination (Eq.1) of the normal (σ_n_) and shear (σ_s_) stress, with respect to the tensile (S_t_), compressive (S_c_) and shear (S_s_) strength (Hoffman 1967; Zelle et al. 2011).

1 $$ \begin{array}{l}{\sigma}_n\ \ge\ 0\ \to\ FI=\frac{1}{S_s}{\sigma}_s+\frac{1}{S_t}{\sigma}_n\\ {}{\sigma}_n < 0\ \to\ FI=\frac{1}{S_t{S}_c}{\upsigma}_{\mathrm{n}}^2+\left(\frac{1}{S_{\mathrm{t}}}-\frac{1}{S_{\mathrm{c}}}\right){\upsigma}_{\mathrm{n}}+\frac{1}{{\mathrm{S}}_{\mathrm{s}}^2}{\upsigma}_{\mathrm{s}}^2\end{array} $$

An FI above 100% would indicate instant failure. Finally, 4) the strain energy density (SED) distribution in the bone was analysed to assess the stress shielding effects. As demonstrated in many bone remodelling studies a relative increase in SED would indicate stimulation of bone growth, a reduction would induce bone resorption (Ghosh and Gupta 2014; Van Lenthe et al. 2002; Lerch et al. 2012). Excessively high SED values would be associated with bone failure (Mirzaei et al. 2014).

## Results

The results presented were chosen at the moments at which peak stresses occurred in the femoral implant, which coincided with heel strike (HS), mid-stance (MS) or toe-off (TO). At these moments, the contact forces were the highest, representing the most detrimental instances in the simulated gait cycle.

### Stresses within the femoral component

**Fig. 2:**
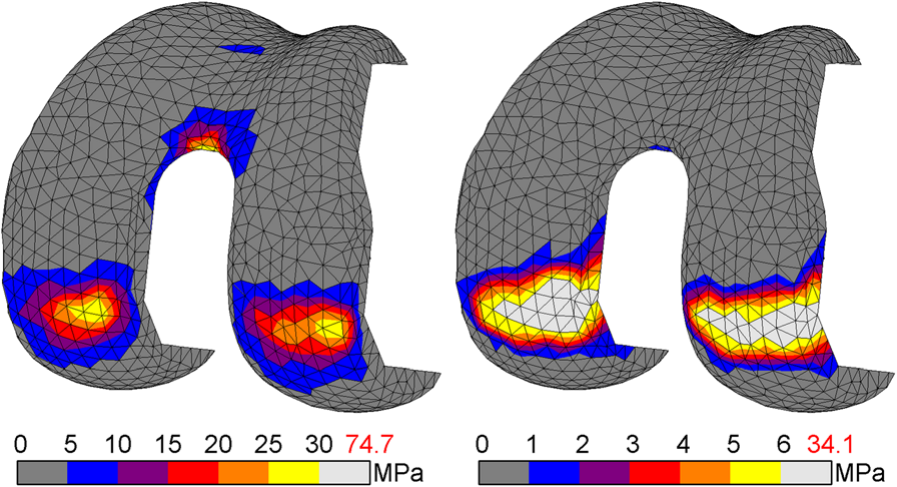
Compressive stress for CoCr (l) and PEEK (r) femoral component. Stresses exceeding 5% of respective yield stress (600 MPa *vs.* 117 MPa) are coloured *white*, with the peak stress depicted in *red*

**Fig. 3:**
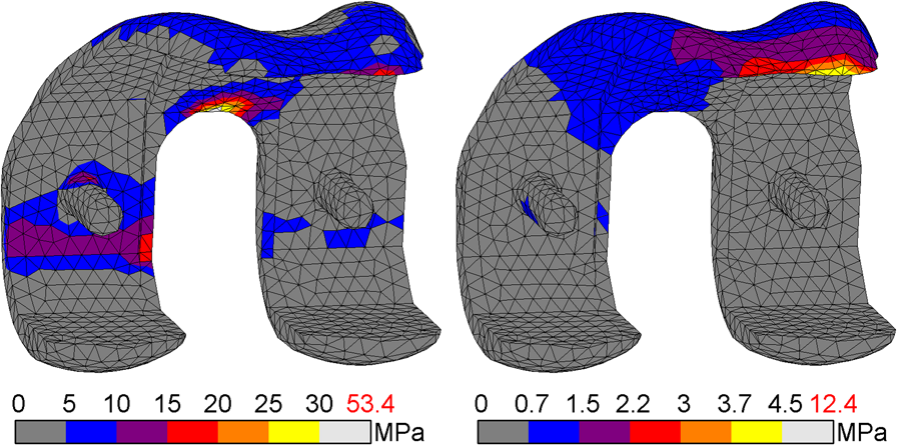
Tensile stress for CoCr (l) and PEEK (r) femoral component. Stresses exceeding 5% of respective yield stress (600 MPa *vs.* 90 MPa) are coloured *white*, with the peak stress depicted in *red*

Changing CoCr to PEEK altered the stress patterns found in the femoral component notably. The CoCr device showed compressive stress intensities at the tibiofemoral contact site and the intercondylar notch, which were of similar magnitude. In the PEEK component the stress intensities at the contact site were also present, but no intensity was present at the notch site (Fig. 2). At the internal surfaces the location of the most prominent CoCr tensile stresses was closely related to the compressive patterns exteriorly, with the exception of an additional intensity at the tip of the anterior flange. The higher PEEK tensile stresses all accumulated in the anterior flange (Fig. 3).

The peak compressive stresses within the components were 75 MPa and 34 MPa for CoCr (TO) and PEEK (MS), respectively, which was well below the compressive yield stress of 600 and 117 MPa. Peak tensile stresses reached up to 53 and 12 MPa for CoCr (HS) and PEEK (TO), respectively. Considering the yield stress of both materials, PEEK was more at risk in compression than CoCr (13 *vs.* 29% of yield stress), and similarly loaded in tension (13 *vs.* 9% of yield stress). As a result, the factor of safety for PEEK was lower under compression, but similar in tension.

### Stresses within the bone cement

**Fig. 4:**
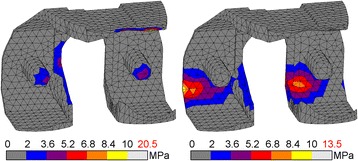
Compressive stress for CoCr (l) and PEEK (r) bone cement. Stresses exceeding 10% of yield stress (97 MPa) are coloured *white*, with the peak stress depicted in *red*

**Fig. 5:**
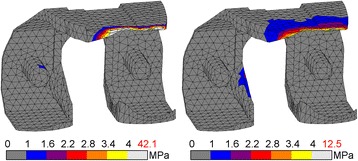
Tensile stress for CoCr (l) and PEEK (r) bone cement. Stresses exceeding 10% of yield stress (40 MPa) are coloured *white*, with the peak stress depicted in *red*

Distinct differences were present in the bone cement below the femoral component. The stiff CoCr component transferred compressive loads mainly in the pegs and cement pocket faces perpendicular to the applied force, away from the tibiofemoral contact site. The highest compressive cement stress occurred during toe-off with 21 MPa (Fig. 4). Tensile stresses at that moment reached up to 42 MPa at the tip of the anterior flange (Fig. 5). During the entire gait cycle the cement mantle supporting the PEEK implant was loaded predominantly in the posterodistal area, with no other distinct regions of stress intensities. The peak compressive stresses occurred during mid-stance (14 MPa). The peak tensile stresses were found at heel strike (13 MPa). Cement stresses were well below PEEK/CoCr compressive yield stress (14 *vs.* 21%). The cement mantles experienced up to 33 and 105% of tensile yield stress for PEEK and CoCr respectively. This resulted in a higher factor of safety for PEEK in both compression and tension.

### Assessment of cement-implant interface failure

The distribution of compressive stresses acting on the interface was similar to that occurring in the bone cement. For the CoCr implant, the highest compressive stress was 23.8 MPa, at toe-off, whilst PEEK generated the highest compressive stress of 15.6 MPa at mid-stance. The total surface area that underwent compression during the gait cycle was largest in CoCr, but generally at a low stress level, in contrast to PEEK, where the surface area was smaller, but with higher stress levels.

The tension patterns suggest that the implant ‘opened up’ during gait. When the component was loaded distally under compression (during stance phase), the flanges parallel to the loading direction (i.e. the posterior condyles and anterior flange) were moving outward, away from the bone. In both implants this phenomenon occurred, albeit to a different extent. The total surface area in the PEEK construct undergoing tension was higher than for CoCr. However, the loads were distributed over a larger area, resulting in lower stress levels. The CoCr interface was loaded in tension in the most proximal region of the posterior condyles and, distinctly, in the proximal region of the anterior flange. The maximum tensile stress for the PEEK implant was 0.48 MPa, at heel strike, and 0.85 MPa for the CoCr implant, at toe-off.

There was also a pronounced difference between the shear interface stress pattern around the PEEK and CoCr implants. In the CoCr construct all areas parallel to the loading direction underwent relatively high shear stresses. The most affected was the anterior flange, which peaked at 2.2 MPa at toe-off. In the model with the PEEK implant, shear stresses were mainly found in the distal interface and posterior chamfers areas. The highest shear stress around the PEEK implant of 1.4 MPa occurred during mid-stance.

**Fig. 6:**
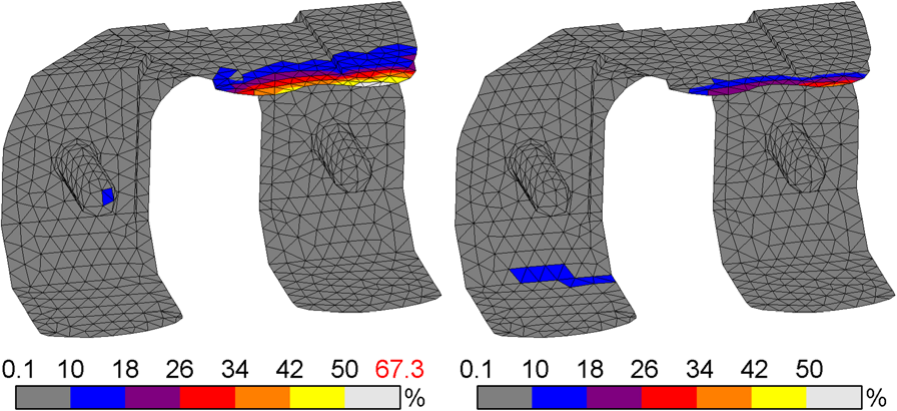
Hoffman failure criterion for CoCr (l) and PEEK (r) cement-implant interface. Peak FI is depicted in red

Failure patterns did not change throughout gait for each implant, but did differ between implants. For CoCr the maximum FI was found during toe-off (FI = 67%). PEEK showed a peak FI of 45% at heel strike. With both CoCr and PEEK the affected area was concentrated at the proximal anterior flange, where also tensile and shear stresses mainly occurred (Fig. 6). These data suggest a higher factor of safety for the PEEK construct interface compared to CoCr.

### Strain energy density levels in the periprosthetic bone

**Fig. 7:**
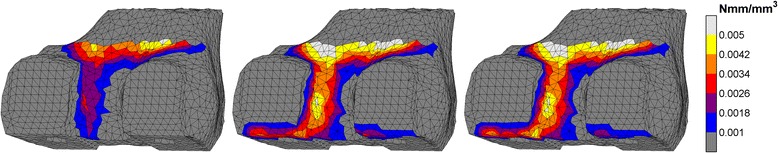
CoCr (l), PEEK (m) and ‘intact’ (r) strain energy density in the periprosthetic femur

Relative to the ‘intact’ case, SED levels around the CoCr implant were lower and expanded over a smaller amount of the bone volume during the gait cycle. Higher levels of SED were located in the medial intercondylar notch and extended above the condyles to the entire width of the posterior periprosthetic femur. This pattern recurred in the PEEK configuration. Importantly, additional regions of higher SED were found in the posterior chamfers of the condyles and extended throughout the bone to the proximal section of the periprosthetic femur (Fig. 7). Both CoCr and PEEK peaked similarly with 0.014 and 0.016 Nmm/mm^3^, respectively. The total amount of strain energy was different, with the PEEK strain energy accumulating more distally. Hence, the SED pattern was more similar to the simulated intact case than the one generated around the CoCr implant (Fig. 7).

**Fig. 8:**
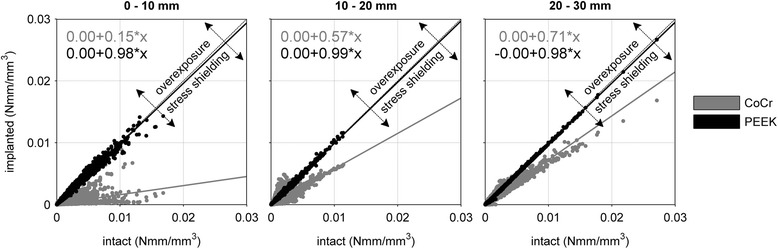
Correlation of strain energy densities between an ‘intact’ reference and de CoCr and PEEK reconstructions in 10 mm thick slices from distal to proximal periprosthetic femur at toe-off

Element-by-element comparisons between the three cases at toe-off emphasized the strong correlation between ‘intact’ and PEEK throughout the entire periprosthetic region; in three 10 mm sections the slope of the least-squares curve (0.98, 0.99 and 0.98) is near-ideal (Fig. 8). Stress shielding in the CoCr reconstruction is most obvious in the distal 10 mm section with a slope of 0.15. This improved towards the metaphysis (0.57) but remained lower than PEEK after 30 mm (0.71). The slopes showed statistically significant differences (p < 0.05) in all sections.

## Discussion

Overall we observed that the PEEK construct generated different stress/strain patterns in all areas of interest for this study. The loads were passed on throughout the reconstructed knee in a manner that appeared to be closer to a physiological situation than a CoCr device (Dickinson et al. 2012).

The potential failure mechanisms for the implant and cement mantle were different between the devices. PEEK reduced the factor of safety in compression, while in tension the factor of safety for implant, cement mantle and cement-implant interface was increased. Previous studies earlier highlighted the dominant role of tensile loads in failure of reconstructions with metal knee implants (Culleton et al. 1993; Gruen et al. 1979; Harper and Bonfield 2000; Stolk et al. 2004; Zelle et al. 2014). This implies PEEK may be beneficial with regard to tensile loads under gait loading conditions. However, the PEEK and implant-cement interface stress levels may increase with body-mass index (BMI), more demanding activity levels, or sub-optimal fixation, which have not been investigated here. The mechanical survival of polymer femoral components has previously been demonstrated in a clinical follow-up study by Moore et al. (1998), who published on a polyacetal femoral component in combination with a UHMWPE tibial component. After 10 years of postoperative follow-up none of the cemented implant failures were attributed to implant fracture, aseptic loosening or wear of the femoral component (Moore et al. 1998). Polyacetal has mechanical properties similar to PEEK, implying that the reduced factor of safety for the implant as found in the present study, should be sufficient to accommodate the risks of implant fractures (Thompson et al. 2001).

Comparison of the bone strains between both components and the surrogate intact case suggests that the more compliant PEEK indeed reduces stress shielding. Bone strains were more similar between intact and PEEK than between intact and CoCr. The most distinct differences were found at the distal chamfers, where strains were very low in the CoCr construct compared to PEEK and the intact bone, as suggested in several previous studies (Akay and Aslan 1996; Bobyn et al. 1992; Gillies et al. 2009; Lee et al. 2012). Although bone adaptation was not simulated in the current study, the current remodelling stimulus patterns coincide with areas of bone loss as reported in the literature (Järvenpää et al. 2014; Lavernia et al. 2014; Van Lenthe et al. 2002).

The model that was utilized for this study was limited to level gait as for time-dependent parameters, such as bone remodelling and fatigue, normal walking is considered most determinative (Van Lenthe et al. 1997; Morlock et al. 2001). For construct integrity assessment, however, higher demanding activities may be more appropriate, such as squat or stair ascend. In the current model most properties of the cement-implant interface for CoCr were adopted from literature (Zelle et al. 2011). The properties of the PEEK-PMMA interface were assumed to be similar to that of CoCr, although it is expected that the interface stiffness will be lower, and likely also the strength in tension. These parameters should be determined further in an experimental setup, after which they can be incorporated in the current model to investigate their effect on the structural integrity of the PEEK femoral reconstruction. Likely, the difference in interface properties will affect the stress magnitudes, but the relative stress distributions will be very similar to was has been simulated. Thirdly, for the comparison with an intact knee, we did not recreate the intact geometry and kinematics of the knee, but only changed the material properties. Obviously, the femoral geometry does not align exactly with the natural bone. This means that cortical bone is not always mapped to the contacting areas and softer elements will alter the natural stress distribution in those areas. Towards the periprosthetic region the mismatch will be smaller and compliant elements will have encountered stiffer elements to pass on the stresses, cancelling out part (but not all) of the influence of mismatched Young’s moduli. We decided to use the same mesh and map CT properties to the construct to avoid influence of differences in mesh geometries, just to see the effect of the change in Young’s modulus, and whether or not the PEEK representation is likely to be closer to the intact situation. It is likely that the comparison that was made for stress shielding will be altered, but we do expect the same trend that was observed in this study. In addition, the SED distributions as determined in the current study only represent the initial condition. To study the changes in bone density, a remodelling simulation is required, preferably incorporating additional loading scenarios besides normal walking.

## Conclusions

Our aim was to provide insight into the femoral stress distribution when changing the material of the femoral component from CoCr to PEEK. We wanted to investigate whether a PEEK implant would be a feasible solution to relevant issues in current applications. Therefore, as a minimum requirement, we started with the most relevant activity: level walking. This study indicates that during a standard ISO gait cycle the performance of the PEEK femoral component is not inferior to the CoCr implant and that it can reduce the periprosthetic stress shielding. If potential harm of a new implant is excluded during preclinical research, less adverse events can be expected in clinical use. Patients can then benefit from the improved mechanobiological aspects PEEK can deliver over conventional TKA devices.

## Abbreviations

CoCr: Cobalt-Chromium
CT: Computed tomography
FI: Failure index
HS: Heel strike
MS: Mid stance
PEEK: Polyetheretherketone
SED: Strain energy density
TKA: Total knee arthroplasty
TO: Toe off
UHMWPE: Ultra-high molecular weight polyethelene
